# Nanoparticle penetration and transport in living pumpkin plants: *in situ *subcellular identification

**DOI:** 10.1186/1471-2229-9-45

**Published:** 2009-04-23

**Authors:** Eduardo Corredor, Pilar S Testillano, María-José Coronado, Pablo González-Melendi, Rodrigo Fernández-Pacheco, Clara Marquina, M Ricardo Ibarra, Jesús M de la Fuente, Diego Rubiales, Alejandro Pérez-de-Luque, María-Carmen Risueño

**Affiliations:** 1 Centro de Investigaciones Biológicas, (CIB) CSIC, Ramiro de Maeztu 9, E-28040, Madrid, Spain; 2 Instituto de Nanociencia de Aragón, Universidad de Zaragoza, Edificio Interfacultativo II, Pedro Cerbuna 12, 50009, Zaragoza, Spain; 3 Instituto de Ciencia de Materiales de Aragón (ICMA)Departamento de Física de la Materia Condensada, CSIC-Universidad de Zaragoza Pedro Cerbuna 12, 50009, Zaragoza, Spain; 4 Instituto de Agricultura Sostenible, CSIC, Alameda del Obispo s/n, Apdo, 4084, E-14080, Córdoba, Spain; 5 School of Biosciences, University of Birmingham, B15 2TT Birmingham, UK; 6 Centro de Biotecnología y Genómica de Plantas, Universidad Politécnica de Madrid, ETS Ingenieros Agrónomos, Ciudad Universitaria s/n, 28040, Madrid, Spain

## Abstract

**Background:**

In recent years, the application of nanotechnology in several fields of bioscience and biomedicine has been studied. The use of nanoparticles for the targeted delivery of substances has been given special attention and is of particular interest in the treatment of plant diseases. In this work both the penetration and the movement of iron-carbon nanoparticles in plant cells have been analyzed in living plants of *Cucurbita pepo*.

**Results:**

The nanoparticles were applied *in planta *using two different application methods, injection and spraying, and magnets were used to retain the particles in movement in specific areas of the plant. The main experimental approach, using correlative light and electron microscopy provided evidence of intracellular localization of nanoparticles and their displacement from the application point. Long range movement of the particles through the plant body was also detected, particles having been found near the magnets used to immobilize and concentrate them. Furthermore, cell response to the nanoparticle presence was detected.

**Conclusion:**

Nanoparticles were capable of penetrating living plant tissues and migrating to different regions of the plant, although movements over short distances seemed to be favoured. These findings show that the use of carbon coated magnetic particles for directed delivery of substances into plant cells is a feasible application.

## Background

Nanobiotechnology was born as a hybrid discipline, a combination of biotechnology and nanoscience. In recent years, nanoparticles with sizes typically below 100 nm, have been applied in several fields of bioscience and biomedicine, with an increasing number of commercial applications [1]. Advances have been made in the field of biomedicine, including the development of tools for pathogen bio-detection, tissue engineering and MRI contrast enhancement [2]. Special interest have been focused on those applications developed for targeted delivery of substances and drugs, implying direct movement of nanoparticles to specific organs [3-5].

The possibility of targeting the movement of nanoparticles to specific sites of an organism paves the way for the use of nanobiotechnology in the treatment of plant diseases that affect specific parts of a plant. Different procedures have made use of nanoparticles in plants, such as the controlled release of bioactive substances in solid wood [6-8] and plant transformation through bombardment with gold or tungsten particles coated with plasmidic DNA [9]. In recent years, a breakthrough has been made as a result of Torney *et al. *[10] who were able to control the intracellular release of substances into protoplasts using mesoporous silica nanoparticles. Despite these advances, the delivery of nanoparticles into plant tissues has been limited to methods involving bombardment [9,10], a methodology that does not allow massive application of particles in large numbers of plants, thus being of little use for agronomic purposes.

Recently, our group has applied carbon-coated iron nanoparticles to pumpkin plants in order to develop tools for the directed release of chemicals into plant organs susceptible to infection by pathogens that specifically attack them [11]. Using different microscopy methodologies to monitor their presence in plant tissues, we have shown that these nanoparticles penetrate living plant tissues. But as the foregoing observations are only a first step in the directed distribution of nanoparticles in living plants, to what extent these particles are capable of migrating properly to reach their target has yet to be established

The aim of this work was to analyse the penetration and movement of nanoparticles in plant cells, and the capacity of a magnetic field to retain them in specific parts of the plant. Two different application methods were used: injection, and spraying, the latter being a more practical option from an agronomic standpoint. Correlative microscopy, on both light and electron microscopy levels, was used as a convenient experimental methodology, providing evidence of intracellular localization of nanoparticles and their displacements from the application points. Long range movement of particles through the plant body were also detected, these particles being found near one of the magnets used to immobilize and concentrate them.

## Methods

### Plant material and growth conditions

Pumpkin (*Cucurbita pepo *L.) plants were selected for preliminary experiments due to their large vessel size, which would facilitate the transport of nanoparticles through the vascular system. Plants were grown using a polyethylene bag system as previously described [11,12]. A strip (11 × 28 cm) of glass fibre paper (Whatmann GF/A) was inserted into a polyethylene bag (25 × 35 cm) as physical support for the development of the root and as a way for the nutrient solution to rise by capillary action and reach the whole root. Pumpkin seeds were germinated in Petri dishes with moistened filter paper. Fifteen day old pumpkin seedlings having root lengths of about 4–5 cm were transferred to the bag, placing them on the upper side of the system. Twenty ml. of Hoagland nutrient solution [13] was added to each bag, and refilled later when necessary. The bags were suspended vertically in boxes and the plants were grown in a controlled environment chamber with a day/night temperature of 20 ± 0.5 8C, a 14 h photoperiod and a photosynthetic photon flux density of 200 μmol m^-2 ^s^-1^.

Some nanoparticle-treated plants and some control plants were placed in pots to evaluate whether the treatment would have any effect on the subsequent growth of the plants.

### Nanoparticle synthesis

Carbon-coated iron nanoparticles were produced by the discharge arc method [14,15] based on that previously designed by Krätschmer-Huffman [16]. The Krätschmer-Huffman method uses a cylindrical chamber, in which there are two graphite electrodes: a stationary anode containing 10 μm starting iron powders, and a moveable graphite cathode. An arc is produced between the graphite electrodes in a helium atmosphere. The graphite electrode is sublimed and builds up a powder deposit (soot) on the inner surface of the chamber. It was in this material that we found: carbon nanostructures, amorphous carbon and iron, and iron oxide nanoparticles encapsulated in graphitic layers, together with a small amount of non-coated or partially coated metallic particles.

These particles (which are not biocompatible) were eliminated by chemical etching, washing the soot with 3 M HCl at 80°C. This procedure favours the formation of carboxylic groups on the graphitic shell, which, due to their hydrophobic nature, contribute to the stability of the final particle suspension. In order to eliminate the amorphous carbon and thereby increase the concentration of magnetic nanoparticles, the powder underwent magnetic purification. To do this, stable suspensions of the particles were prepared in a surfactant solution: 2.5 g of SDS in 500 ml of distilled water. A field gradient produced by a permanent magnet (maximum magnetic field 3 KOe) was used for magnetic separation of this suspension. Employing this method, only the carbon-coated magnetic nanoparticles are attracted by the magnet whereas the amorphous carbon remains in the supernatant, which is subsequently eliminated.

### Nanoparticle application

Nanoparticles were suspended in gelafundine, a commercial succinated gel (30 g/l gelatine, 0.45% w/v sodium chloride, 0.21 g/l calcium, Braun, Melsungen-Germany), and the solution was kept in water in an ultrasonic bath for several minutes. This solution was selected because it had been used as a convenient suspension medium for nanoparticles in experiments with animals. A biocompatible magnetic fluid was obtained in this way, which was used for the inoculation of plants.

**Figure 1:**
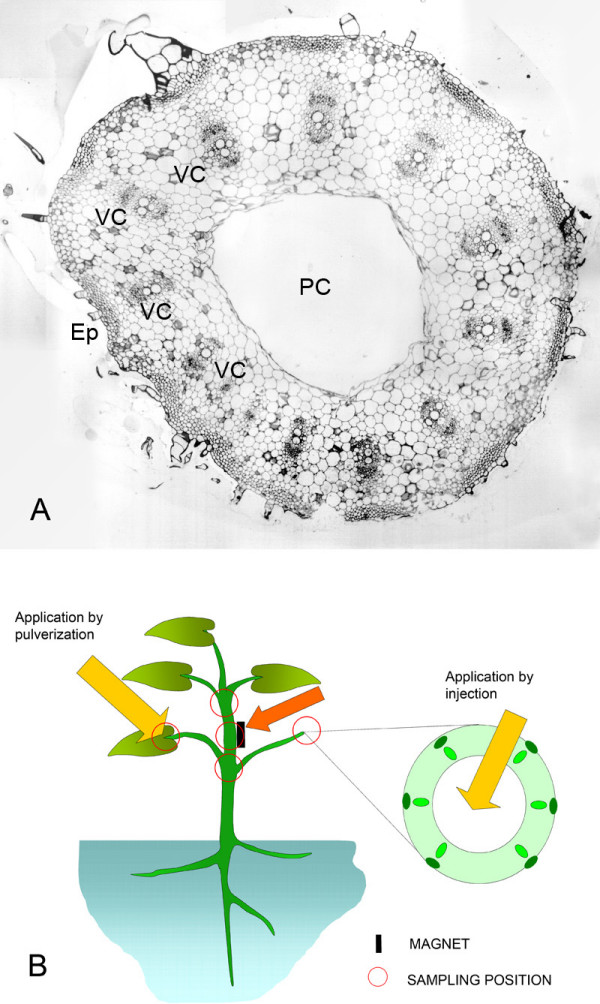
**Cellular organization of the pumpkin stem, and experimental design of nanoparticle application and sample collection**. A) Transversal semithin section of pumpkin stem after staining with toluidine blue, showing the main structural elements of the stem: Vascular cores (VC), epidermis (Ep), pith cavity (PC). B) Diagram showing the bioferrofluid application points, the position of the magnet in the plant and the sampling points.

To favour the penetration of the nanoparticles into the plant tissues, the bioferrofluid was injected into the pith cavity of the leaf petiole (Fig 1A) (Application by injection; Fig 1B). A methodology closer to the one to be employed by agronomist and breeders in field applications was also checked, which consisted of placing droplets of the ferrofluid on the leaf surface, close to the insertion point of the petiole, to facilitate the penetration of the particles into the tissues of the petiole, in this way emulating the effect of spraying a nanoparticle solution onto a cultivated plant (application by spraying; Fig 1B). In order to evaluate the possibilities of a directed transport of the nanoparticles to specific regions of the plant, small magnets were placed in other positions on the plant (illustrated in Fig. 1B) such as the petiole of the leaf opposite the injection point.

### Collection and processing of samples for microscopy analysis

Stem and petiole samples from selected positions on the plant were collected 24, 48 and 168 hours after application of the bioferrofluid and processed for microscopic analysis as previously described [17]. The selected positions (shown in fig. 1B) were the application points and points situated close to the magnets, before and after them, according to the movement that the particles were supposed to follow. They were fixed in Karnovsky solution (4% formaldehyde + 5% glutaraldehyde, in 0.025 M cacodilate buffer, pH 6.9) for 4 h at room temperature, and then kept in the buffer at 4°C.

Approximately 1 mm-thick, hand-cut cross sections of the fixed samples were dehydrated through an ethanol series, infiltrated and embedded in Epon resin (Serva, Heidelberg, Germany).

### Microscopy analysis methods

For correlative microscopy, 1–2 μm sections were cut from the polymerised blocks, observed on a light photomicroscope (Leitz, Germany) under phase contrast, bright field and dark field to identify the presence of nanoparticle aggregates as previously described [11] and photographed using an Olympus DC10 digital camera. The regions of interest were trimmed and 70–100 nm ultrathin sections were obtained. The sections were counterstained with 5% uranyl acetate for 30 min, rinsed in bidistilled water, dried and observed on a transmission electron microscope Jeol 1010 at 80 kV. Micrographs were taken with a Megaview digital camera (Gatan).

## Results

Correlative microscopy was used to score the presence of nanoparticles in samples collected from different positions, close to the application points and magnet locations. Following the procedures previously established [11], a first level of analysis was performed by examining 1–2 μm Epon sections on the light microscope in search of nanoparticles. Aggregates of nanoparticles appear as dark, optically dense signals under bright field, that correlate with a refringent signal under dark field and phase contrast [11]. To make a precise identification of the presence of nanoparticles by light microscopy, their presence was only considered as proven when the three microscopies employed (phase contrast, bright field and dark field) provided correlated images (see additional files 1, 2, 3: correlative images 1, 2, 3 for the micrographs used to select the nanoparticle-containing cells to be analyzed at TEM). For correlative microscopy, those areas that appeared to contain nanoparticles were selected for analysis under TEM.

Two different application methods were employed, namely, injection and spraying (see MM). Plant samples were collected 24 h, 48 h and 168 h after application, these time periods having been previously determined to be appropriate for detectingt internalization of nanoparticles into the tissues [11].

### Internalization of nanoparticles close to the application point

#### a. Application by injection

**Figure 2:**
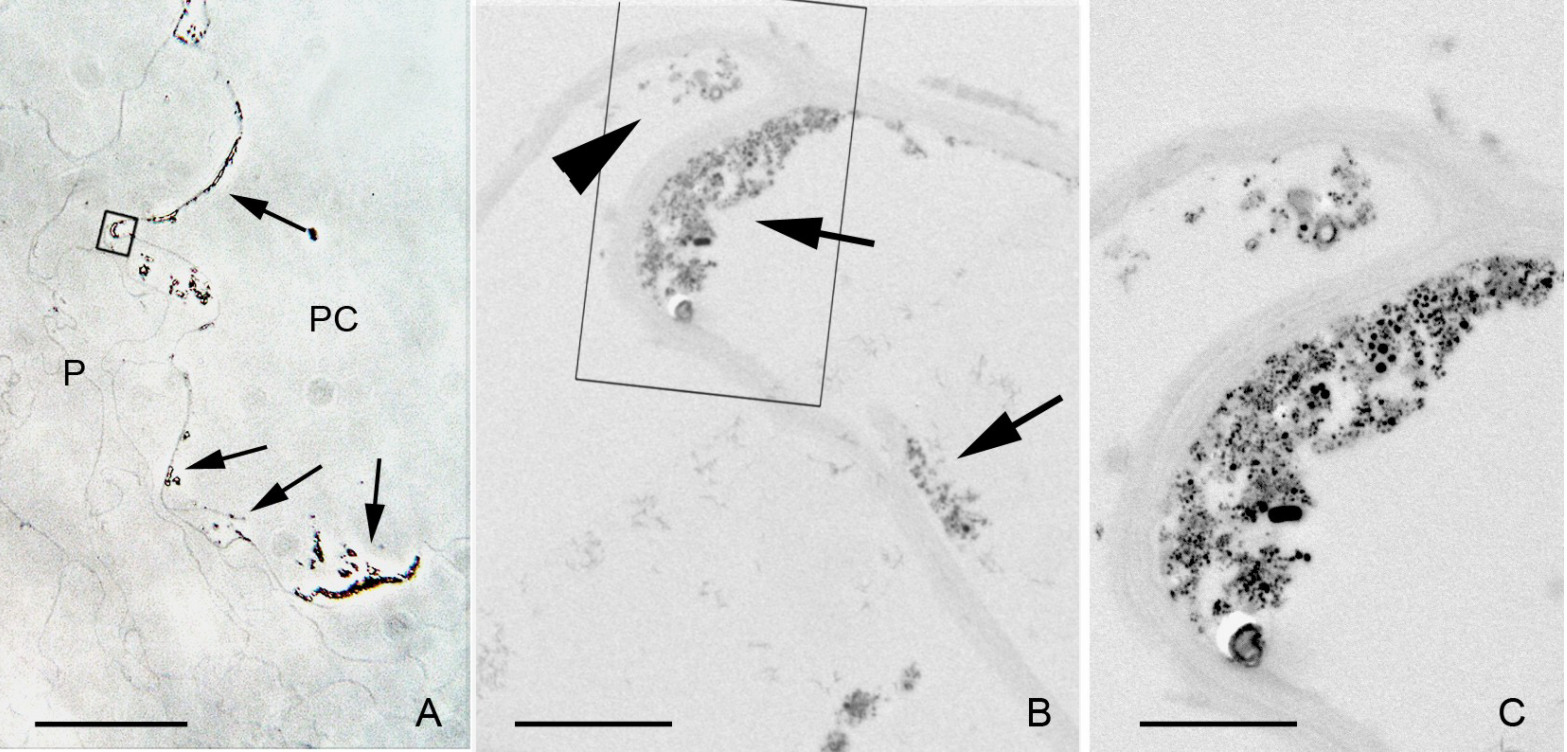
**Penetration of nanoparticles into the first cell layer surrounding the pith cavity**. A) Phase contrast image of the parenchymatic cells (P) closer to the pith cavity (PC). The nanoparticle aggregates on the application surface appear as an optically dense material (arrows). B) Transmission electron micrograph of the region squared in (A). Nanoparticle aggregates appear in the cell wall facing the pith cavity (arrows) and into the cytoplasm of the first cell layer (arrow head). C) High magnification of the region squared in (B). The intracellular aggregate is smaller than the extracellular one in the pith cavity. Bar in A = 40 μm; B = 2 μm; C = 1 μm.

**Figure 3:**
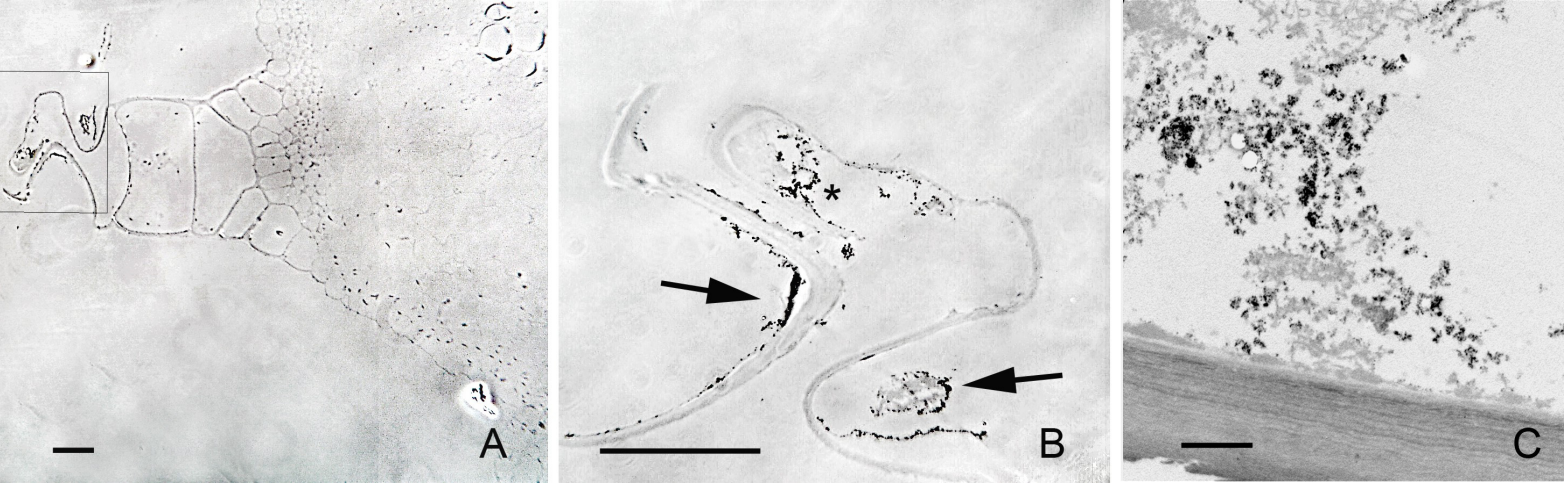
**Nanoparticles migration to the apex of a trichome**. A) Phase contrast image of a trichome. B) Detail of the region squared in (A), showing the presence of nanoparticle aggregates as optically dense material, in the cell interior (*) as well as in the outer face of the epidermal cell wall (arrows). C) TEM image of a region equivalent to the one indicated by (*) in (B). Nanoparticles appear as aggregates associated to intracellular structures. Bar in A and B = 50 μm; C = 2 μm;.

Samples of the stem collected 24 h after nanoparticle application showed a dense, dark precipitate in the inner surface of the pith cavity (Fig. 2A, and additional file 1: correlative images 1), which could even be observed with the naked eye. This precipitate was composed of nanoparticles, as assessed by electron microscopy (Fig. 2). The areas where nanoparticle deposits were identified were selected for analysis by correlative microscopy. When these regions were observed under TEM, nanoparticles could be clearly identified in such aggregates, as well as their presence in the parenchimatic cells surrounding the pith cavity (Figs. 2B, C). These nanoparticles appeared in the form of intracellular aggregates, much smaller than the ones observed in the pith cavity. In these samples, the presence of nanoparticles in the epidermis of the stem, especially those associated with trichomes, were also detected. The trichomes revealed the presence of nanoparticles in the cytoplasm and on the outer side of the cell wall (Fig. 3 and additional file 2: correlative images 2).

**Figure 4:**
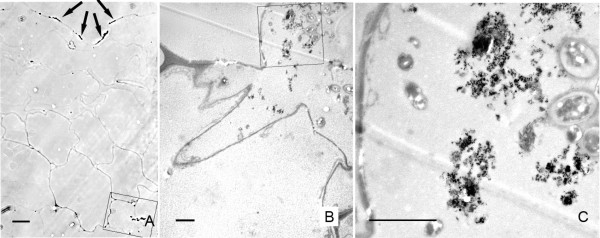
**Penetration of nanoparticles into individual cells of the parenchyma**. A) Phase contrast image showing the relative position of the cells carrying nanoparticles (squared) respect to the location of nanoparticle deposits in the pith cavity (arrows). B) TEM image of a region equivalent to the one squared in (A). Nanoparticles are electron dense, and appear surrounded by organelles. C) High magnification of the region squared in (B). Nanoparticles are aggregated. The cellular structures close to them show bright white inclusions. Bar in A = 20 μm; B and C = 2 μm.

**Figure 5:**
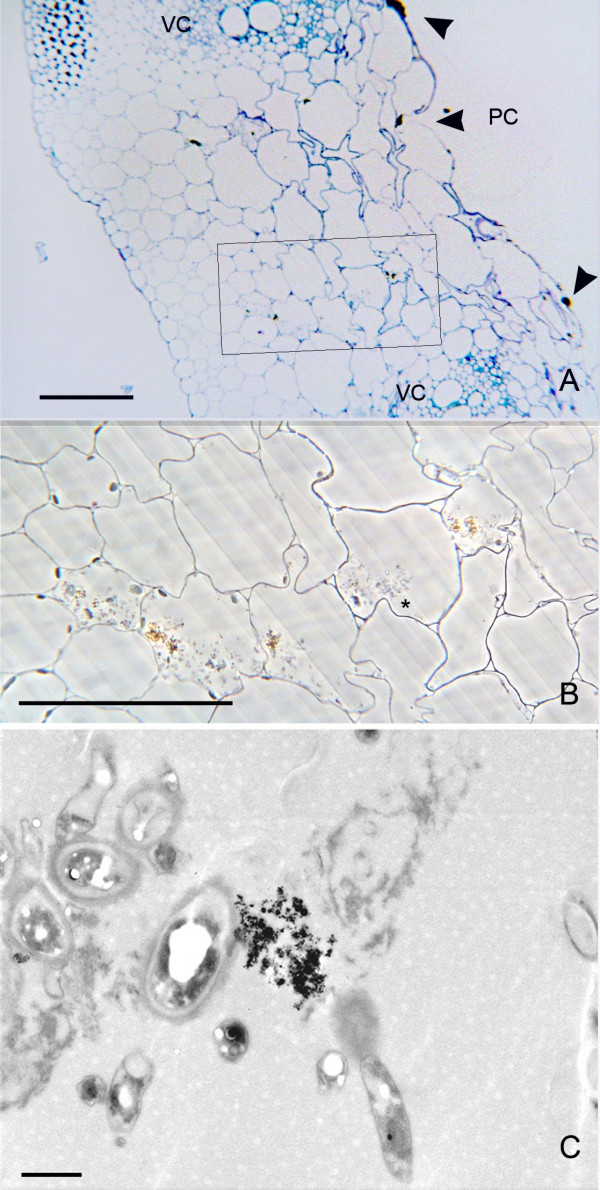
**Presence of nanoparticles in adjacent parenchymatic cells**. A) Bright field image, toluidine blue staining of a section of the stem showing cells carrying nanoparticles (squared area) between two vascular cores (VC). The nanoparticle deposits in the cell wall facing the pith cavity (PC) appear labelled with arrowheads. B) Phase contrast image of a section consecutive to the one showed in (A), with a detail of the region squared in (A). Dark material appears in the cell cytoplasm surrounded by dense structures. The surrounding cells do not show such a dense cytoplasm. C) TEM image showing a detail of a nanoparticle aggregate inside one of the cells showed in (B) which is labelled by (*). Bar in A and B = 100 μm; C = 1 μm.

In samples collected 48 h after application, light microscopy observations showed that the nanoparticles had migrated towards the interior of the stem parenchyma. The aggregates in the inner surface of the pith cavity still remained there, likewise aggregates of nanoparticles in individual parenchymatic cells could be observed. (Fig. 4A and additional file 3: correlative images 3) The presence of such aggregates in the cytoplasm of the cells was accompanied by the presence of more numerous cytoplasmic structures compared to the surrounding cells. This higher density in the cytoplasm was detectable even under phase contrast microscopy, which showed the cell cytoplasm carrying the nanoparticles with a granular structural pattern, while in the surrounding cells there were no signs of these structures (Figs. 4A, B). Under TEM observation, the nature of these cytoplasmic structures was diverse with many of them containing large inclusions of starch that exhibited their typical clear aspect under TEM (Figs. 4C, 5C). The nanoparticles appeared to be freely aggregated in the cytoplasm, although the conditions employed for sample fixation were not optimized for membrane preservation and contrasting, and the possibility that they were in a subcellular compartment cannot be excluded.

**Figure 6:**
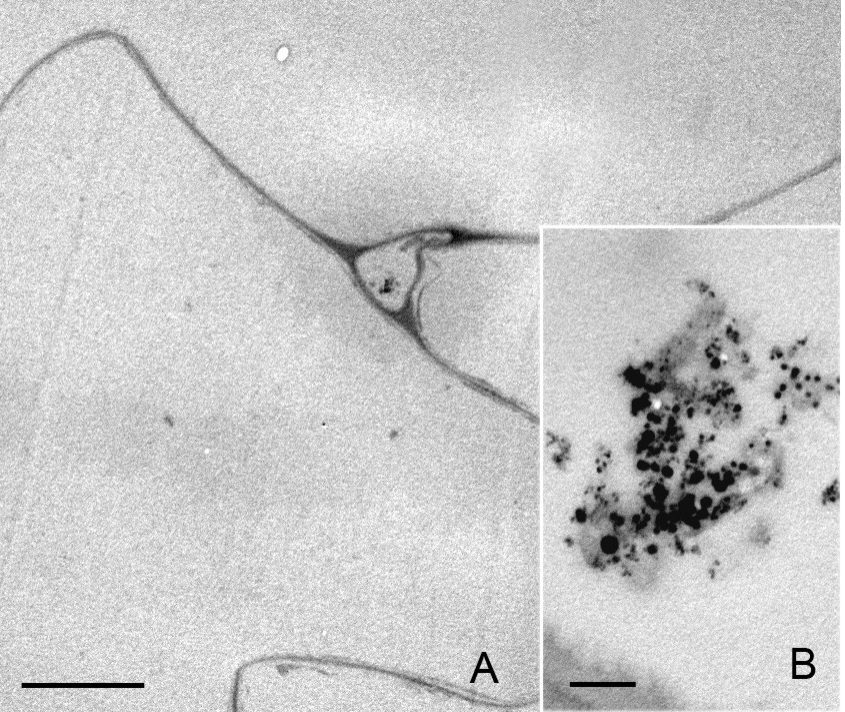
**Ultrastructural imaging of nanoparticles in the extracellular space**. A) image showing three confluent parenchymatic cells with a triangle shaped extracellular region between them. The parenchymatic cells do not show traces of presence of nanoparticles. B) High magnification of the extracellular channel shown in (A) displaying nanoparticle aggregates. Bar in A = 10 μm; B = 200 nm

These nanoparticle-carrying cells not only appeared as individual cells in the parenquima, but "chains" of several adjacent parenchymatic cells (2 to 5) were also frequently observed carrying nanoparticle aggregates and with the same higher density in the cytoplasm (Figs. 5A, B). These linear groups of cells appeared in positions between vascular cores, having no apparent relationship with the vascular system, and oriented radially to the stem surface (Fig. 5A). In the same way as in individual cells containing particles, the TEM analysis revealed nanoparticle aggregation in clusters (Fig. 5C) surrounded by a high density of intracellular components in these groups of parenchymatic cells. In the same samples, it was also possible to identify the presence of small aggregates of nanoparticles in intercellular spaces (Fig. 6). When detected, these intercellular areas were not associated with any nanoparticle presence in neighbouring cells (Fig. 6A).

**Figure 7:**
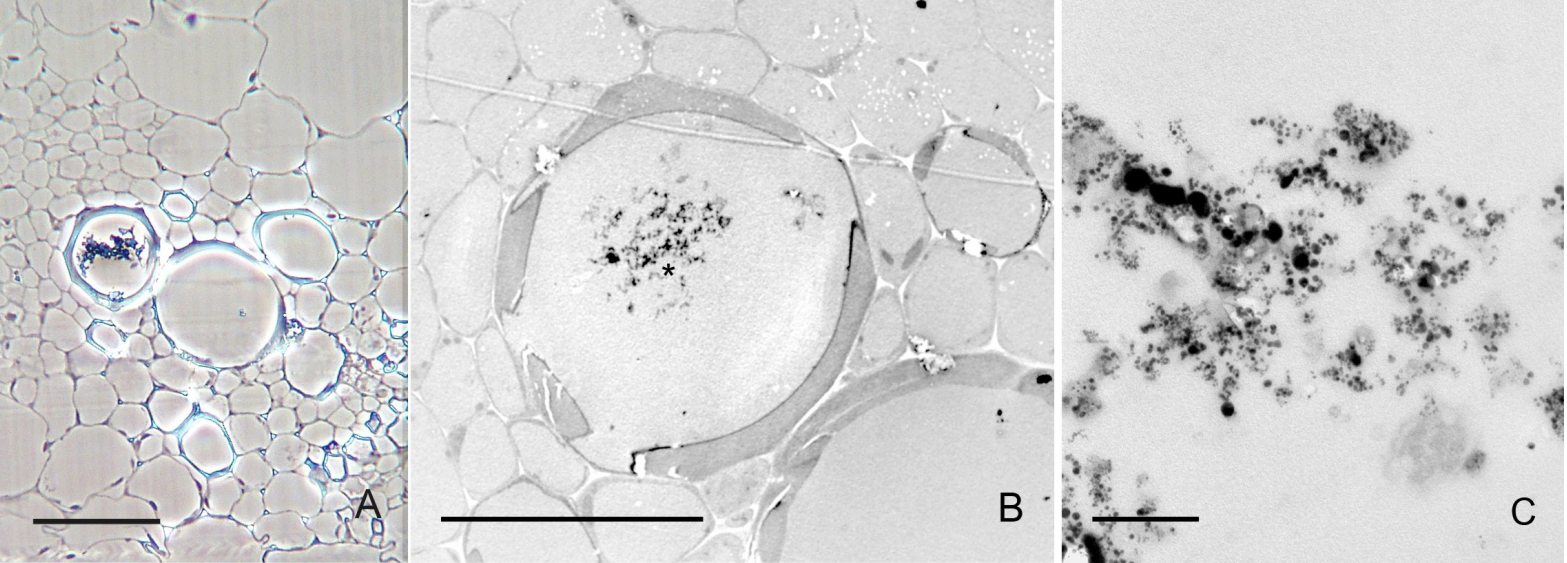
**Nanoparticle localization inside the xylem vessels**. A) Phase contrast image of a vascular core, showing a cluster of dark bright dense material inside of a xylem vessel. B) TEM image of the vessel containing the dense material in an ultrathin consecutive section. C) Detail of the region indicated in (B)(*), showing presence of nanoparticles in the electron dense material. Bar in A and B = 30 μm; C = 500 nm.

Interestingly, nanoparticle aggregates were also detected in the tissues surrounding the application point, inside the xylem vessels, 48 h after application by injection, (Fig 7). Correlative microscopy permitted us observe that the aggregates in the interior of the vessel (Fig. 7A and additional file 4: correlative images 4) corresponded with associations of nanoparticles of variable sizes (Fig. 7C). Samples collected 168 h after application by injection showed no remarkable nanoparticle deposits in the pith cavity, nor in any other part of the stem.

#### b. Application by spraying

**Figure 8:**
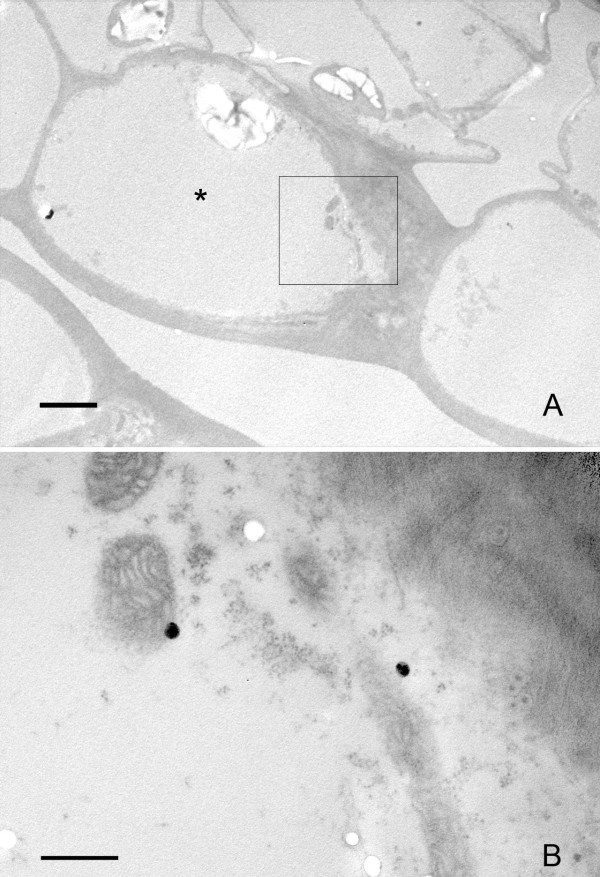
**Penetration of nanoparticles in the epidermal cells after application by pulverization**. A) Low magnification TEM micrograph showing the epidermal cells. The cell carrying nanoparticles appear labelled by an asterisk (*). The cytoplasm does not show any special feature compared to the underlying parenchymatic cells. B) detail of the region squared in (A), showing the presence of two nanoparticles as dark electron dense spots. Bar in A = 3 μm; B = 500 nm.

After spraying, it was not possible to detect the presence of nanoparticles by light microscopy in samples ranging from 24 to 168 h after application. However, when samples collected 168 h after application were analysed under the electron microscope, the presence of nanoparticles was identified in cells from the epidermis of the petiole close to the application point. (Fig. 8). In these cells the nanoparticles appeared isolated, not in aggregates, and the densities of intracellular structures observed in the host cell were similar to those in neighbouring cells. It was also noted that no nanoparticles were clearly detected in cells beyond the first epidermal layer. Neither were nanoparticles detected in other stem samples collected 24 or 48 h after application.

### Internalization of nanoparticles in locations far from the application point

**Figure 9:**
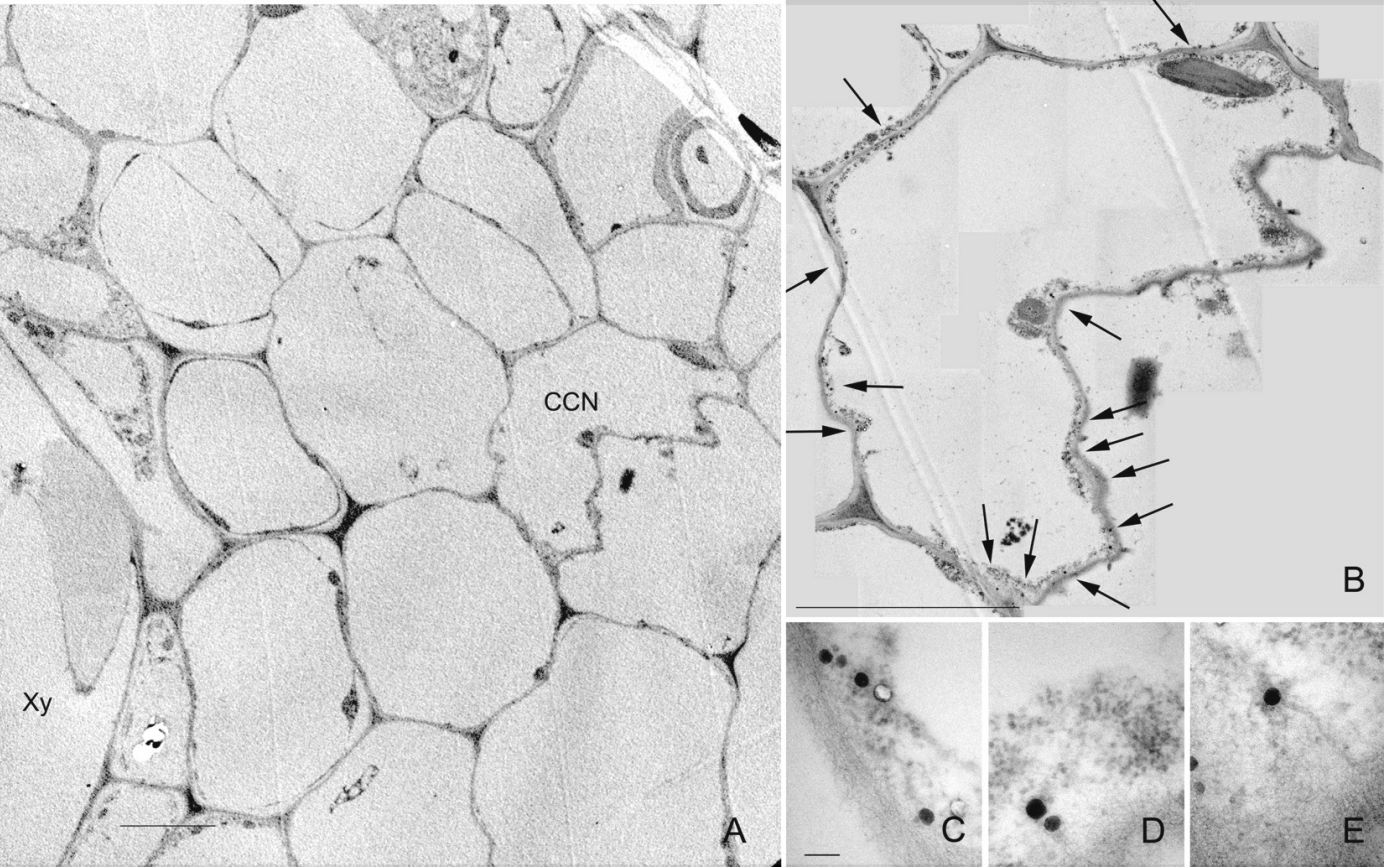
**Migration/transport of nanoparticles to individual cells close to the magnet location**. A) Low magnification TEM micrograph, showing the position of the cell carrying nanoparticles (CCN) respect to a xylem vessel (Xy). The CCN does not show any special differential features in the cytoplasm in comparison with the neighbouring cells. B) High magnification image of the CCN showing the location of several nanoparticles (arrows). C, D, E) Details of some isolated nanoparticles of those arrowed in (B). Bar in A and B = 5 μm; C = 100 nm.

Light microscopy observations did not detect nanoparticles in samples at positions far from the application point, or near magnet locations, either before or after the magnetic device. Direct analysis of these samples was carried out by TEM, selecting for the analysis the vascular cores and the surrounding cells, given that these were expected to form part of the route for long range transport in plants. In these areas, the presence of particles was detected only occasionally in individual cells close to the xylem (Figs. 9A–B) and in samples near the magnet collected 48 h after their application by injection. The particles appeared isolated in the cytoplasm, without any clear association among them in most cases (Figs. 9C–E). The diameters of the particles present in a single section of the cytoplasm of one cell were measured and showed a mean diameter of 46.7 ± 1.7 nm (n = 18). There were no apparent differences in the cytoplasm content of the host cell with respect to that of the neighbouring cells. No particles were detected after exhaustive screening of ultrathin sections from samples collected from positions far from the application point and distant from the magnet, irrespective of whether the application was by injection or spraying.

To evaluate whether the application of nanoparticles had any effect on the subsequent growth of the plants, some treated plants were planted in pots and kept under controlled conditions until the flowering period. No apparent differences were observed between plants subjected to the treatment and the control plants, the former even being able to produce fruit which indicated that treatment with nanoparticles did not provoke a toxicological effect on plant growth. At tissue level, the structural organization of the stem samples of treated and non-treated plants was similar, with no signs of damage at light and electron microscopy levels. Only the cells containing the nanoparticle agglomerates exhibited more dense cytoplasms, as previously stated. This fact suggested that the penetration of nanoparticles through the tissues did not damage them.

## Discussion

In the present work, intracellular penetration of carbon-coated iron nanoparticles applied *in planta *was tracked using correlative microscopy, including a first screening of the samples with optical microscopy (bright field, dark field and phase contrast) followed by analysis through transmission electron microscopy. This strategy has allowed us to confirm both the progressive penetration of particles through the plant tissues and their presence in the form of intracellular aggregates, when injected into tissues near the application site, in as short a time as 48 h. Furthermore, 48 h after injection, isolated nanoparticles in the cytoplasm of individual cells close to a vascular core were observed in the proximity of a magnetic device located far from the application point. When applied by spraying, isolated nanoparticles were also found in the cytoplasm of epidermic cells, in regions near the application point.

### Cell and tissue response to the presence of nanoparticles

Nanoparticles move away from the application point, on the interior of the stem, where they were injected, to the outer epidermis through the tissues. The fact that a dense cytoplasm with starch-containing organelles was observed concomitantly with nanoparticle aggregates in the cytosol, suggests that plant cells could respond to the presence of a high density of nanoparticles by changing their subcellular organization. Results also showed that the nanoparticles had already appeared in the outer surface of the plant, both inside and outside of the trichomes, 24 h after application, indicating that at least part of the bioferrofluid can be expelled in a short time. Several examples of cytotoxicity of carbon nanoparticles have been described in various animal systems [18-23] as well as different effects derived from the application of magnetic ferrofluid [24,25]. Cytotoxicity has also been associated with the dose of nanoparticles [26]. This correlation between the number of nanoparticles and cytotoxicity agrees with our results, which showed that no subcellular rearrangements were detected in those plant cells in which only a few isolated nanoparticles were detected (Figs. 8 and 9), compared with the response detected in cells carrying aggregates of nanoparticles. But, to date, there has been no description of changes in cell architecture and organization after nanoparticle internalization in vivo, so it is not possible to assess if the observed reaction is specific to our material or if it is a general trait of cell reaction to high concentrations of nanoparticles within the cell. Moreover, the solution used for nanoparticle suspension, namely gelafundine, contains calcium, an important second messenger in plants. The possibility that some calcium remains attached to the carbon coat of the nanoparticle and could have an effect on cell response, does not seem to be very plausible due to the chemical properties of the nanoparticle surface (hydrophobic, not negatively charged...), even so, it cannot be completely excluded.

### Internalization of nanoparticles near the application point

The observation of particle aggregates in adjacent parenchymatic cells suggests a movement from cell to cell. Another question to be addressed is the presence of nanoparticles as intracellular aggregates, as it seems quite unlikely that nanoparticles have the capability of entering cells other than as individual particles. The mechanism involved in this aggregation is unclear. As indicated in MM, the carbon-iron nanoparticles employed show a tendency to aggregate in aqueous solutions because of the chemical characteristics of the carbon coat. Aggregation of nanoparticles is a common phenomenon [27], so it is possible that this reclustering takes place spontaneously after individual internalization into the cytoplasm, although it could not be discarded that the nanoparticles were redirected by the cell to a specific subcompartment or region in the cytoplasm.

The presence of nanoparticles in epidermal cells after application by spraying is of special interest. As stated before, one of the main drawbacks of other methods is that they cannot be employed for agronomic purposes. The method used in this work resembles the procedures which would be used by breeders and coordinators of phytosanitary control, employing both large scale and hand-on spraying to leaf surfaces. The fact that nanoparticles passed through the epidermal cell wall opens up the possible application of these nanotechnology tools for agronomical purposes. Given the special characteristics of the epidermic outer cell wall, specifically its considerable thickness, and the presence of protective waxes, a possible particle penetration point could be through the stomata and the subestomatic chambers. In fact, this aperture is a route used by pathogens of different species, such as the white pine blinter rust [28]. Interestingly, water-suspended 43 nm hydrophilic particles have been described as occasionally penetrating *Vicia faba *leaves through stomatal pores [29]. Recently, the uptake of magnetite nanoparticles through the root system of *Cucurbita maxima *plants was reported, though a significant uptake of particles was only found in plants growing in liquid media [30]. So, in order to make the system suitable for agronomical purposes, methodological improvements would need to be made.

### Long range transport and putative movement through the vascular system

The main reason for developing the carbon-iron nanoparticles and their application in this work was to accumulate them at a specific site in the plant by means of magnetic fields applied specifically to a certain area. As shown in Fig. 9, isolated nanoparticles were detected in the cytoplasm of cells close to the vascular core, far from the application point and near the magnet. The position of these cells suggests that the route taken by the particles involved the use of the plant vascular system. Direct observation of freshly cut material revealed the presence of bioferrofluid, specifically in the interior of the xylem vessels [11], along with nanoparticle aggregates, 48 h after application (Fig. 7) suggesting that the particles can use this system for long range transport. It has to be noted that those particles found far from their application point (Fig. 9) were quite homogeneous in size, around 46 nm in diameter on average, when compared with the variable sizes found in the original mixture detected in the aggregates in the pith cavity and cells close to the application point. (Figs. 2, 3, 4, 5, 6, 7). This may suggest that a certain critical size is required for the appropriate movement of particles through the plant by long range transport mechanisms.

## Conclusion

Taken together, the results reported here demonstrate that the carbon-iron nanoparticles are able to get into the cells of living plants, and move to remote positions, possibly through the vascular system. These facts constitute an important step forward in elucidating the mechanisms of interaction between plant cells and nanoparticles and thus, in designing strategies for using nanoparticles for targeted delivery of substances. In this sense, methodological improvements are required to make the system suitable for agronomical purposes, one of them being the functionalization of the nanoparticles with organic groups that can help their penetration into the phloem or make their internalization into cells more efficient. The evidence reported here, that nanoparticles were able to move to locations where the magnets were located, far from the application point, would suggest the potential use of carbon-coated nanoparticles for directed delivery of substances for phytosanitary purposes, using magnetic fields to retain the particles in areas of interest.

## Authors' contributions

EC carried out the electron microscopy study, the correlative light and electron microscopy analysis and wrote the first manuscript draft. PST participated in the experimental design, contributed to the draft and writing of the manuscript and its revision, and participated in the coordination of the work. MJC and PGM carried out the processing of plant samples, the correlative light microscopy analysis under different visualization modes and some electron microscopy essays. RFP carried out the synthesis of nanoparticles and the bioferrofluid suspension. CM and MRI participated in the design of the nanoparticle synthesis and preparation of the suspension, and in the design of the study. JMF contributed to the experimental design of nanoparticle synthesis and to the writing of parts of the manuscript. DR participated in the design of the study and helped in experiments of nanoparticle treatments to the plants. APL designed and carried out the nanoparticle treatments to the plants, and helped in the writing of some parts of the manuscript. MCR conceived the study, participated in the design and coordination of the work and helped to draft the manuscript. All authors read and approved the final manuscript.

## Supplementary Material

Additional file 1 **Correlative images 1**. Bright and dark field visualization of the nanoparticles shown in Figure 2A: A) Bright field image, of the same field in (Fig. 2A). The nanoparticle aggregates appear as a dark material. B) Dark field image of the same field in (Fig. 2A). The nanoparticles appear as bright refringent. Bar = 40 μm Click here for file

Additional file 2 **Correlative images 2**. Bright and dark field visualization, and electron microscopy of the nanoparticles shown in Figure 3B. A) Dark field image, B) Bright field image, C) Electron micrograph of the nanoparticle aggregates of (A) and (B). Bar in A and B = 50 μm; C = 2 μm. Click here for file

Additional file 3 **Correlative images 3**. Detail of the cell squared in fig 4A. A) Phase contrast image, showing nanoparticle aggregates that are visible as an intense dark material. The cytoplasm appears dense and displaying numerous structures and organelles in comparison with the surrounding cells. B) Same field that in (A), bright field image. Bar = 20 μm. Click here for file

Additional file 4 **Correlative images 4**. Bright and dark field visualization of the nanoparticles shown in Figure 7A. A) bright field image. B) dark field image. Bar = 30 μm. Click here for file
