# Supplementary figures and images for: Nanoparticle penetration and transport in living pumpkin plants: in situ subcellular identification
Source: BMC Plant Biol. 2009 Apr 23;9:45. doi: 10.1186/1471-2229-9-45 (PMC2680855; doi:10.1186/1471-2229-9-45)

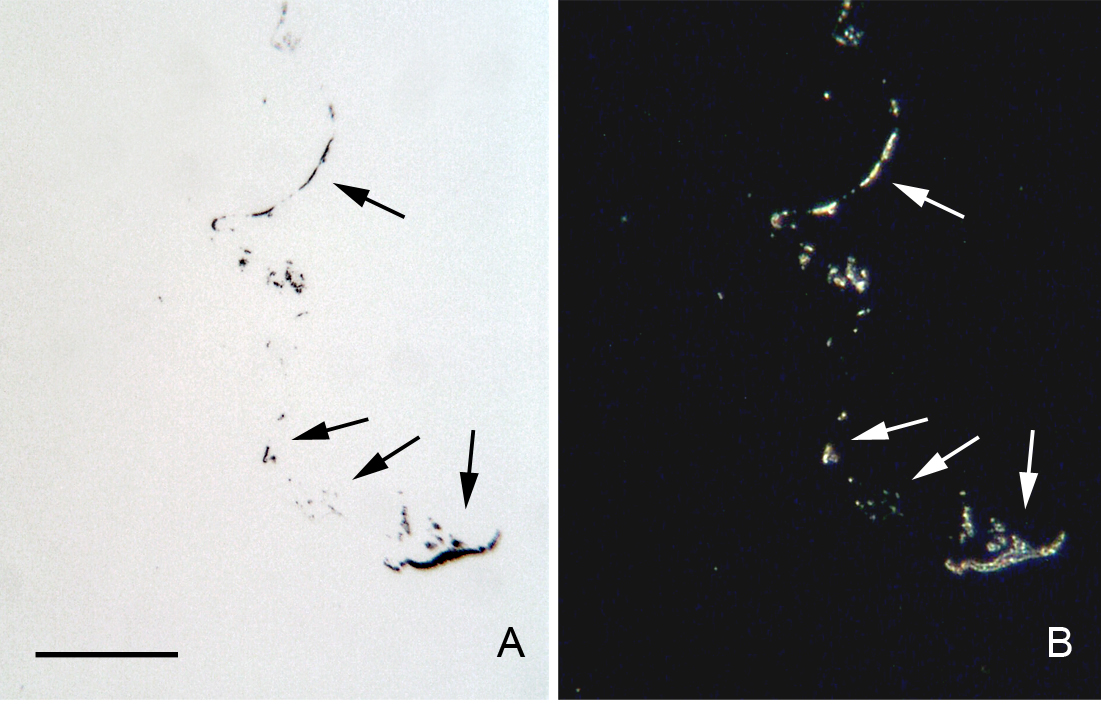

Supplement: Additional file 1 — Correlative images 1. Bright and dark field visualization of the nanoparticles shown in Figure 2A: A) Bright field image, of the same field in (Fig. 2A). The nanoparticle aggregates appear as a dark material. B) Dark field image of the same field in (Fig. 2A). The nanoparticles appear as bright refringent. Bar = 40 μm [file 1471-2229-9-45-S1.jpeg]

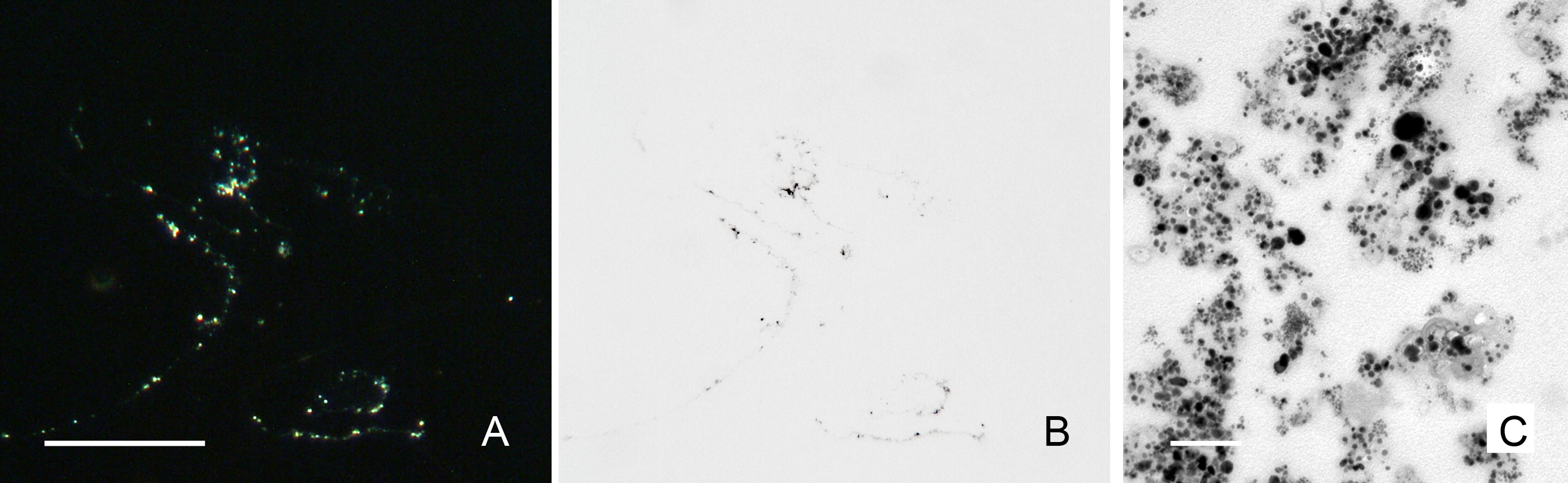

Supplement: Additional file 2 — Correlative images 2. Bright and dark field visualization, and electron microscopy of the nanoparticles shown in Figure 3B. A) Dark field image, B) Bright field image, C) Electron micrograph of the nanoparticle aggregates of (A) and (B). Bar in A and B = 50 μm; C = 2 μm. [file 1471-2229-9-45-S2.jpeg]

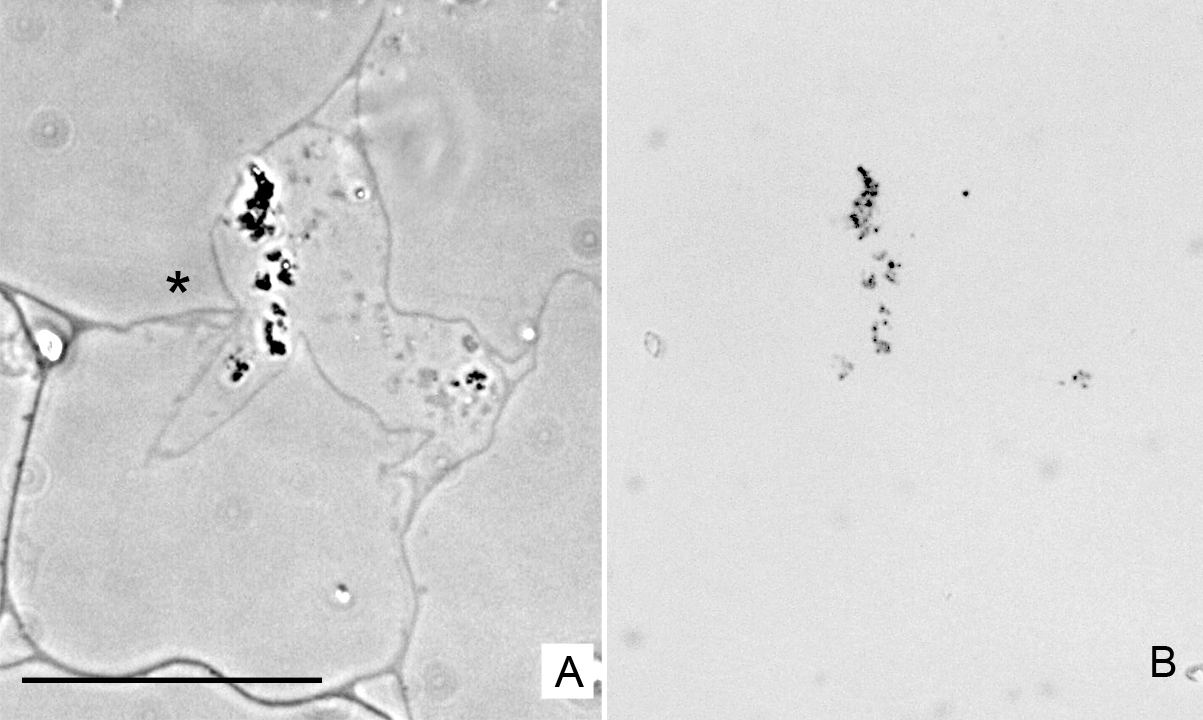

Supplement: Additional file 3 — Correlative images 3. Detail of the cell squared in fig 4A. A) Phase contrast image, showing nanoparticle aggregates that are visible as an intense dark material. The cytoplasm appears dense and displaying numerous structures and organelles in comparison with the surrounding cells. B) Same field that in (A), bright field image. Bar = 20 μm. [file 1471-2229-9-45-S3.jpeg]

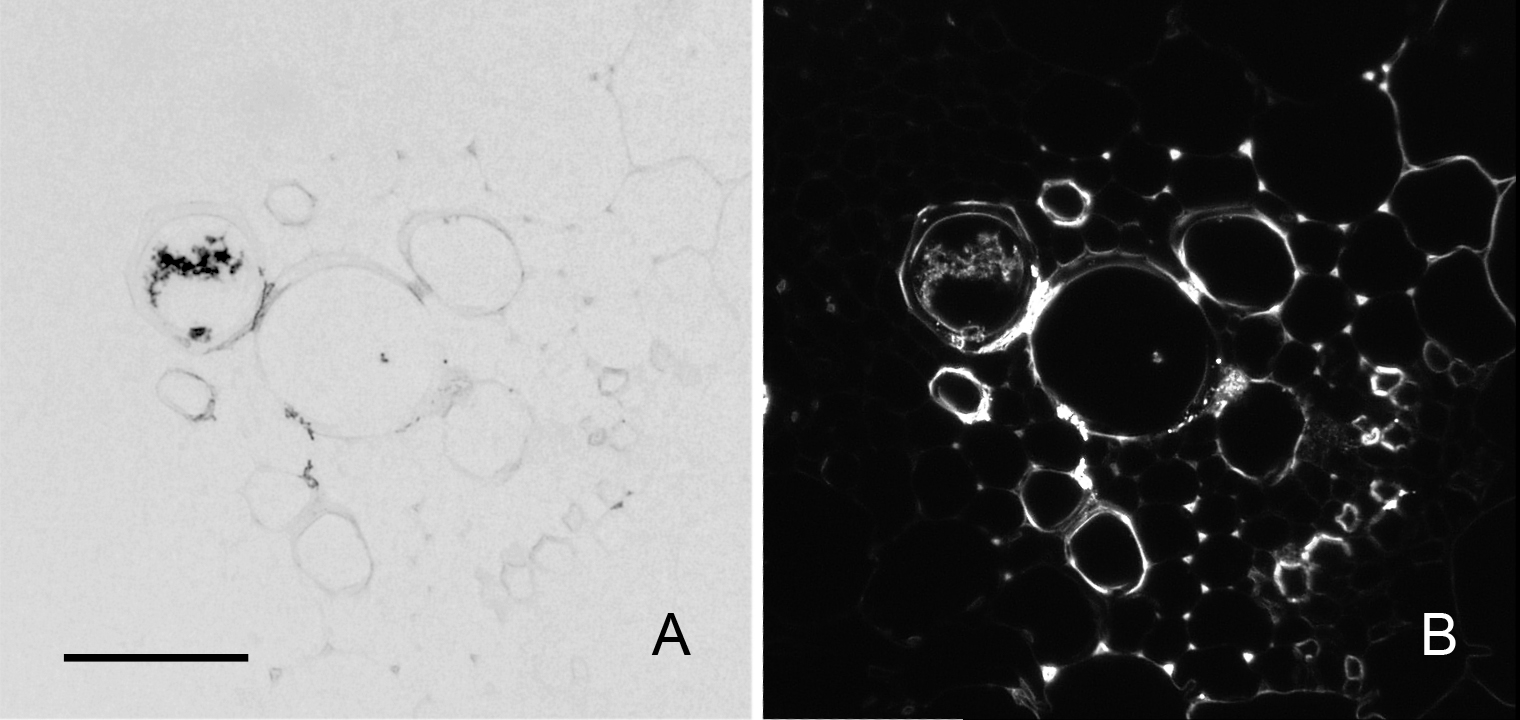

Supplement: Additional file 4 — Correlative images 4. Bright and dark field visualization of the nanoparticles shown in Figure 7A. A) bright field image. B) dark field image. Bar = 30 μm. [file 1471-2229-9-45-S4.jpeg]
